# Facilitators and barriers to healthy eating in a worksite cafeteria: a qualitative study from Nepal

**DOI:** 10.1136/heartasia-2017-010956

**Published:** 2017-10-26

**Authors:** Archana Shrestha, Prajjwal Pyakurel, Abha Shrestha, Rabin Gautam, Nisha Manandhar, Elizabeth Rhodes, Dipesh Tamrakar, Biraj Man Karmacharya, Vasanti Malik, Josiemer Mattei, Donna Spiegelman

**Affiliations:** 1 Department of Epidemiology, Harvard TH Chan School of Public Health, Boston, Massachusetts, USA; 2 Department of Community Medicine, BP Koirala Institute of Health Sciences, Dharan, Nepal; 3 Department of Community Programs, Dhulikhel Hospital – Kathmandu University Hospital, Dhulikhel, Nepal; 4 Laney Graduate School, Emory University, Atlanta, Georgia, USA; 5 Department of Community Medicine, Kathmandu University, Dhulikhel, Nepal; 6 Department of Cardiology, University of Washington, Seattle, Washington, USA; 7 Department of Community Medicine, Kathmandu Hospital, Dhulikhel, Nepal; 8 Department of Nutrition, Harvard TH Chan School of Public Health, Boston, Massachusetts, USA; 9 Department of Global Health and Population, Harvard TH Chan School of Public Health, Boston, Massachusetts, USA

**Keywords:** education, public health, primary care

## Abstract

**Objective:**

Worksite interventions can serve as a potential platform for translating existing knowledge of diabetes prevention and facilitate healthy food choices. The study explored perceptions about healthy eating as well as potential facilitators and barriers to healthy eating among employees in a wire manufacturing factory in Nepal.

**Methods and materials:**

We conducted a cross-sectional exploratory qualitative study in a wire manufacturing industry in eastern Nepal. We conducted three focus group discussions (FGDs) with a total of 26 employees and four in-depth interviews (IDIs) with cafeteria operators/managers from a wire manufacturing factory in eastern Nepal. FGDs and IDIs were audio-recorded, transcribed verbatim and analysed using the thematic method.

**Results:**

Most employees defined healthy eating as the consumption of food prepared and maintained using hygienic practices and fresh foods in general. Major barriers to healthy eating included unavailability of healthy foods, difficulty in changing eating habits, the preference for fried foods in Nepali culture and the high costs of some healthy foods. The most commonly reported facilitator of healthy eating was the availability of affordable healthy food options in worksite cafeterias.

**Conclusion:**

Availability of healthy food options at an affordable price could lead to healthier food choices in the worksite.

## Introduction

The prevalence of non-communicable diseases (NCDs), including in particular that of cardiovascular disease, diabetes and cancer, is on the rise in low-income and middle-income countries.^1 2^ Annually, NCDs are responsible for 16 million premature deaths.^3^ In Nepal, the burden of NCDs, as measured by disability-adjusted life years, has increased alarmingly between 1990 and 2010.^4^ The Stepwise Approach to Surveillance 2013 survey reported that 21% of the adult Nepalese were overweight or obese, 26% had raised blood pressure, 4% had raised blood sugar, 23% had raised total cholesterol, and 99% did not consume the recommended five or more servings of fruits and vegetables a day.^5^

Modifiable risk factors such as sedentary lifestyle, poor diet and excess body weight are reported to have a large effect on the risk of NCDs.^6 7^ An unhealthy diet increases the risk of major non-communicable chronic diseases such as coronary heart disease, stroke, diabetes and some cancers in adults, and indirectly contributes to an increased risk by being overweight and obese.^8 9^ In Nepal, the typical dietary pattern with refined grains, meat and alcohol was associated with a higher prevalence of overweight and obesity.^10^ Deep fried foods were associated with hypertension; the cereal and vegetable pattern was inversely associated with diabetes prevalence.^11^

Worksites, which are communities with their own social networks and infrastructure for disseminating information to employees, may provide a unique opportunity to deliver messages that encourage healthy eating behaviours. Employees spend most of their waking hours at worksites. Well-designed worksite-based health programmes have shown positive impacts on employee health.^12 13^ Environmental changes that support low-cost, healthy food choices, places for physical activity and group-based health education classes have been demonstrated as components of successful worksite interventions.^14 15^

The literature on employees’ beliefs and opinion about healthy eating in the worksite is limited. Devine *et al* 16 reported that employees are aware of the importance of healthy diet and are willing to choose healthy foods if they are tasty, convenient, reasonably priced and of good quality. Some of the barriers to healthy eating in worksites identified by earlier researches include high cost, limited choices and the inavailability of healthy foods,^17^ heavy workload and lack of breaks,^18^ as well as stress-related eating.^19^

Given that each worksite is unique with its own complex environment, we conducted a qualitative study to explore perceptions about healthy eating and understand facilitators and barriers to healthy eating in cafeterias of a wire manufacturing factory in eastern Nepal. Findings from this study will ultimately be used to develop a culturally acceptable and appropriate environmental and individual-level worksite-based intervention for diabetes and hypertension prevention.

## Methods

### Study context

This study was conducted in a wire manufacturing factory in eastern Nepal with about 745 employees. One of the investigators (PP) provides health promotion and preventive services in the factory as a physician. This study was conducted to collect information to assist in the development of a healthy eating intervention in the cafeteria of the factory to prevent type 2 diabetes (T2D) as a part of larger health promotion programme. Informed consent was obtained from all participants.

### Study design

This is an exploratory cross-sectional qualitative study. We conducted focus group discussions (FGDs) with users of the cafeterias, including manual labourers and administrative staff, as well as in-depth interviews (IDIs) with those running the cafeterias, including operators and a factory manager.

### Study setting

Currently, three cafeterias provide food to the factory employees. The first is the *managers’ cafeteria*, which operates in a small kitchen with a traditional cook and serves about 15–20 people per day. The cost of a lunch is about US$67 cents. The only lunch option that the cafeteria provides consists of white rice, lentil soup, pan-cooked whole wheat bread (roti), yoghourt, pickle, and, occasionally, sweets such as rice pudding (*kheer*), dessert (*rabdi*—condensed milk with high sugar) and radish pudding (*gajarko haluwa*). Sunflower oil is used in the cafeteria, which is considered a healthy oil.^20^ The second cafeteria is called the *staff cafeteria*, which is operated by about three cafeteria staff recruited by the factory. The lunch items consist of white rice, lentil soup, vegetables, bottled pickle, yogort, fresh raw cucumber, carrot and radish salad. The foods are cooked on site and each meal costs about US$42 cents. Soybean oil, which is also considered a healthy oil,^21^ is used for cooking in this kitchen. The third cafeteria is called the *labourer cafeteria* and is operated by an external vendor. The foods available in this cafeteria are milk tea, potato chips (boiled and deep fried with chickpea flour), beaten rice (half-boiled and dried rice), instant noodles, *bhujiya* (processed white rice), white rice, lentil soup, seasonal vegetable, pickle and horse gram. Soybean oil is also used as the cooking oil. An average lunch plate with two cups of rice, a cup of lentil soup and a cup of vegetable is 578 calories with 115 g of carbohydrate, 15 g of protein and 6 g of fat.^22^ The average cost of a meal is US$32 cents.

### Focus group discussions

Two FGDs, each with nine participants, were conducted with manual labourers, and one, with eight participants, was conducted with administrative staff, to maintain homogeneity within the groups. Thus, we recruited a total of 26 participants out of the 30 recruited for the FGDs (response rate=90%), randomly selected from a list of employees of the factory and stratified by job type, that is, administrative staff or manual labourer.

Participants reported demographic information (age, gender, education, income), lifestyle behaviours (alcohol, smoking) and presence of chronic diseases (hypertension, diabetes) on a brief questionnaire administered prior to the FGD. The FGDs explored participant perceptions of healthy eating and facilitators and barriers to healthy eating at the worksite. We used a semistructured FGD guide in Nepali language. We pilot-tested the guide with nine participants in the study population. The nine participants were not included in the main study. The questions in the guide covered three main domains: (1) perceptions of healthy and unhealthy eating; (2) facilitators to healthy eating in the worksite; and (3) barriers to healthy eating in the worksite. The moderator asked open-ended questions to the participants about their opinions and probed for in-depth information. The questions included ‘what do you understand by healthy and unhealthy foods?’, ‘what factors affect your food choices?’, ‘what can facilitate you to make healthier choices?’ and ‘what obstructs you from making healthier food choices?’ All the participants were encouraged to share their honest opinions. All FGDs were conducted in Nepali by a native Nepali speaker. Each session began with an introduction that included a brief explanation of the study and ethical considerations about maintaining confidentiality of the participants. The FGDs were conducted in a private room in the factory to ensure confidentiality and facilitate the honest sharing of opinions. The FGDs lasted for 45–60 min and were audio-recorded. We used an iterative process for data collection. After each FGD, the study team debriefed the discussion and identified key themes emerging from the discussion and topics to be explored further, and subsequently revised the guide.

### In-depth interviews

We purposively selected three cafeteria operators from each of the three cafeterias. We also selected a company manager, who serves as a major decision-maker of the cafeterias in the factory. The interviews were conducted to understand the individual perceptions among cafeteria operators and managers about the facilitators and barriers to healthy eating. Each cafeteria operator represents a cafeteria. A manager was chosen because this is the person responsible for decision-making and changes in the cafeterias.

We conducted semistructured IDIs with the cafeteria operators and manager using a pretested interview guide. The goal of these interviews was to obtain information regarding the facilitators and barriers to healthy eating from the cafeteria operators’ and managers’ perspective. The researcher interviewed the participants with open-ended questions regarding their perceptions on healthy eating, facilitators and barriers to healthy eating in the worksite, operational and managerial aspects of the cafeteria, and facilitators and barriers to making changes that promote healthy eating. We asked questions such as ‘What foods are healthy and unhealthy in your cafeteria?’, ‘What changes would you want to see in the cafeteria to make it healthier?’, ‘What factors would facilitate making the healthy changes?’ and ‘What challenges do you anticipate in order to make healthy changes?’ In each case, the interviewer probed for sufficient descriptive information.

The investigators used the iterative process by discussing each interview shortly after it was completed and making suggestions for future interviews, with subsequent interviews probing more deeply into themes emerging in earlier interviews. Each interview was conducted in Nepali in a private room in the factory. Each interview lasted about 1 hour and was audio-recorded. The IDIs and the FGDs were moderated by ArS or PP.

### Data analysis

Audio recordings from the FGDs and IDIs were transcribed verbatim in Nepali by two trained native speakers. The investigator (PP) reviewed the full transcripts and compared them against the recordings. We used inductive coding to allow findings to emerge from frequent, dominant or significant themes inherent in the raw data. Data were analysed using the thematic framework method to identify the themes related to healthy eating, as well as facilitators and barriers to healthy eating in the cafeteria.^23^ The investigators (PP, ArS) read through the transcripts several times to familiarise themselves with the data. The text was then divided into meaningful units, such as phrases and quotes, and the meaningful units were then condensed. The condensed meaningful units were then abstracted and labelled with codes independently by two of the investigators (PP, ArS) using the RQDA software. The various codes were compared on the basis of differences and similarities and sorted into categories. The categories were further discussed by the investigators for identification and formulation of themes and subthemes. An example of the coding, categorising and formulating theme is shown in table 1.

**Table 1:** An example of coding, categorising and formulating themes

Codes	Definition of codes	Subcategory	Category	Theme
Mixed food	Any reference to the combination of two or more types of foods items	Combination of food	Balanced diet	Description of healthy eating
Vegetarian plus non-vegetarian food	Any reference to the combination of vegetarian and non-vegetarian foods			
Nutrients	Any reference to the combination of different nutrients such as carbohydrate, protein, vitamins and fibres	Combination of nutrients		
Fruits	Any reference to fruits or fresh fruit juice, not the sugar-sweetened fruit juice such as brands like ‘Frooti’ and ‘Real juice’	Fruits	Healthy foods	
Greens	Any reference to green leafy vegetables in specific such as spinach, collard and radish leaves, or in general such as ‘saag’ and ‘saag paat’	Vegetables		
Vegetables	Any reference to vegetables other than green leafy ones such as cabbage, cauliflower, eggplant and so on, but not uncooked radish, cucumber and carrot			
Salad	Any reference to uncooked vegetables, especially carrots, cucumber and radish			
Wheat	Any reference to whole wheat products wheat grains and whole wheat roti	Grains and legumes		
Legumes	Any reference to legumes, cooked, fried or soup			
Milk	Any reference to animal milk products	Meat and milk products		
Yoghourt	Any reference to yoghourts such as lassi, curd (dahi) and plain yoghourt drink (mohi)			
Fish	Any reference to fish such as fish soup, fish curry and fried fish			

## Results

### Study population

The characteristics of the FGD participants are shown in table 2. The mean age of the participants was 35 years. All participants were men. Administrative staff has higher income and education level. More labour workers are smokers and alcohol drinkers. The self-reported prevalence of known hypertension was 19% and known T2D was 4%.

**Table 2:** Characteristics of focus group participants in a wire manufacturing factory in Nepal

Characteristics	Administrative staff (n=9) n (%)	Labourers (n=17) n (%)	Total (n=26) n (%)
Age, years, mean (SD)	34.0 (9.9)	35.4 (9.2)	34.9 (9.3)
Male	9 (100)	17 (100)	26 (100.0)
Income (US$)			
Less than US$3 per day	4 (44.4)	16 (94.1)	20 (76.9)
US$3–15 per day	5 (55.6)	1 (5.9%)	6 (23.1)
Education			
Less than high school	1 (11.1)	17 (100)	18 (69.2)
High school or higher	8 (88.9)	0 (0)	8 (30.8)
Alcohol drinking			
Non-drinkers	6 (66.7)	7 (41.2)	13 (50.0)
Drinkers	3 (33.3)	10 (58.8)	13 (50.0)
Smoking			
Non-smokers	6 (66.7)	7 (41.2)	13 (50.0)
Smokers	3 (33.3)	10 (58.8)	13 (50.0)
Vegetarian			
Yes	4 (44.4)	1 (5.9)	5 (19.3)
No	5 (55.6)	16 (94.1)	21 (80.7)
Known hypertension			
Yes	3 (33.3)	2 (11.8)	5 (19.3)
No	6 (66.7)	15 (88.2)	21 (80.7)
Known type 2 diabetes			
Yes	1 (12.5)	0 (0)	1 (3.8)
No	8 (88.9)	17 (100)	25 (96.2)

### Perception of healthy and unhealthy eating

#### Healthy eating

Healthy eating was often described in terms of fresh, home-cooked and hygienically prepared food. To many participants, eating healthy involved a ‘balanced diet’, which was defined as eating a variety of foods including grains, meat and vegetables. Some participants in the admin staff group stated that high-fibre foods are healthy. Fruits and vegetables were the most commonly mentioned healthy foods. Other foods considered healthy were salad, fish, meat, wheat, pan-cooked whole wheat bread (*roti*), milk, juice, lentils and yoghourt. Majority of the participants thought that boiling was a healthier way to cook compared with frying.

Healthy food means a balanced diet…A balanced diet is the most important. A balanced diet means eating different types of foods like greens, vegetables, etc. when we eat everyday.

Healthy foods are fibrous foods; those foods that have protein like whole wheat are better than rice; boiled or steam is better than oil fried.

Participants thought that packaged foods and bottled soda were healthy if they were consumed before the expiration because they have protein, vitamins and minerals as specified on their package, and the government of Nepal approved them for marketing and consumption. Participants consumed bottled soda to make them feel cool in hot weather and hence was said it to be pleasing. The manual labourers also thought that simple Nepali food ‘*saada khaana’* (white rice, lentil soup and vegetables) was healthy because it contained few spices and was freshly prepared.

#### Unhealthy eating

Unhealthy foods were defined as food items that were stored overnight (*baasi)* and unhygienic (eg, contaminated by flies, stored in damp places). Most participants reported that fried food (particularly items containing a lot of chillies) was unhealthy because oil in general was considered unhealthy. The administrative employees named types of foods that they consider unhealthy, such as fried chicken, toast, doughnuts, pizza, burgers, instant noodles and ready-made deep fried foods. In contrast, manual labourers mostly associated unhealthy foods with being unhygienic.

When asked specifically about foods that might contribute to the prevention of T2D, most manual labourers did not know about T2D or how it can be prevented.

What is diabetes? We don’t know.

What causes diabetes? Which foods cause diabetes? What are its symptoms? We don’t know.

What I think the Diabetes disease you are talking about, is not related to food at all.

One participant from administrative staff group said that rice, potato, sweet foods, mutton and pork may cause diabetes, whereas another participant thought that only sweet foods might cause diabetes.

### Facilitators to healthy eating: from the employees’ perspectives

A structured list of barriers and facilitators to healthy eating in cafeterias as perceived by employees is presented in table 3.

**Table 3:** Structured list of barriers and facilitators to healthy eating in cafeterias as perceived by employees of a wire factory in Nepal

Factors	Facilitators		Barriers	
	Employee perspective	Operator perspective	Employee perspective	Operator perspective
Environmental	Availability of healthy foods Management commitment to provide healthy food options in the cafeteria Regular monitoring of cafeteria for availability of healthy foods	Support and commitment from factory management Additional human resources to provide more whole grain options	Unavailability of healthy foods Higher price for healthier foods, especially fruits Lack of breaks during office hours Reuse of cooking oil to save cost	Unavailability of healthy food options in the cafeteria No regular monitoring of cafeteria Higher price for healthier food Poor cafeteria infrastructure to store, clean and preserve food
Individual	Employee and cafeteria operator knowledge on healthy eating food options	Training and education on healthy eating and hygiene to cafeteria staff Employee acceptance to change food habits	Taste preference for fried food	Lack of knowledge on healthy eating Preference for oily, spicy and fried foods
Social			Feel powerless to negotiate with factory management to bring changes in the cafeteria	Low income of employees restricting the offering of healthier but more expensive food

The employees considered the availability of healthy food options to be a major motivator for healthy eating.

In my opinion, green leafy vegetables should be added…there should not be potato in the morning…potato in the evening…there should be vegetables from time to time…there should be less oil and spices…and food should be served hot…that’s what is necessary for us to eat healthy.

Participants commented that management should be committed to providing and maintaining healthier food options in cafeterias. In addition, they expressed that all the stakeholders—employees, cafeteria operators and managers—should be trained in providing healthy foods and in what is healthy eating. The administrative staff specifically emphasised the importance of a committee that comprised representatives of cafeteria users, cafeteria managers and cafeteria operators to regularly meet and monitor the availability of healthy foods in the cafeterias.

The most important thing is that the company (factory management) should look into these issues, first the company (factory management) should pay the attention and commit.

### Facilitators to healthy eating: from the cafeterias’ perspectives

Similar to employee perspectives, operators also thought that it is the responsibility of higher level factory authorities to ensure the availability of healthy foods in the cafeterias and to control the price of foods sold. As stated by one operator:

If the management wants, they can add or remove anything from the cafeteria.

An operator of the labourer cafeteria specifically mentioned the need for additional human resources to provide healthier food options such as pan-cooked whole wheat bread and fruits because it is labour-intensive to cook the bread and to wash and cut fruits. They commented that the administrative staff, as well as manual labourers, need training and education on healthy eating and hygiene. However, the success of these changes also depends on employees accepting the changes.

### Barriers to healthy eating in cafeteria: from employees’ perspective

The main barriers to healthy eating reported by the manual labourers were lack of availability of healthy food options, high cost of healthy food and taste preferences. They emphasised that the labourer cafeteria does not provide healthy options.

The manual labourers were concerned that purchasing healthier options at the other local restaurants would be much costlier (about US$2 per lunch) compared with 37 cents in the labourer cafeteria.

How can cafeteria keep fruits? Where can we find fruit for 37 US cents per meal?

Additionally, the manual labourers reported that adjusting long-term habits of taste will be difficult. The manual labourers and administrative staff expected fried foods in the cafeteria.

One of the major themes that emerged in the discussion was the price of food. The cafeteria users unanimously agreed that the price of the food in the cafeterias was reasonable but the quality of food was low, especially in the labourer cafeteria. However, they suggested that given the low food price, it is difficult to improve the food quality.

When we eat outside, we get good food if we can spend money. Food is better outside (restaurants) than in this cafeteria, they don’t cook good food in cafeteria that is why it is cheaper than outside (restaurants).

The employees unanimously said that reuse of oil lowers quality of food. The manual labourers reported that they are subjected to strict rules and regulations and are not permitted to leave the factory premises during working hours. During the holiday season (October–November), many manual labourers are on leave, so there is additional workload for those who are still working with lunch breaks of limited duration. Due to their low social and economic status, manual labourers felt powerless to have an impact on decisions regarding the foods cooked in the cafeteria.

We are manual laborers. We can’t say anything. We must eat whatever is given to us to make our stomach full, we need to work anyhow.

### Barriers to healthy eating in cafeteria: from the operators’ perspectives

The major barriers to healthy eating reported by management were the price of food, income of employees, lack of knowledge and cafeteria infrastructure. The cafeteria operator said that the factory authority sets the price of the food depending on the income of the employees. Thus, they stated that they have pressure to manage the food items based on the price of lunch previously set by the authority. This leads them to buy the cheapest option, which is often white rice and potatoes. The majority of manual labourers earned a daily wage of about US$3 per day. The price of healthier foods such as fresh fruits, vegetables and salads was too high to be included in the predetermined price.

The main thing is the money.

The cafeteria operators admitted that they did not know much about healthy eating. The cafeteria managers and cooks stated not having received any training related to healthy eating and/or healthy cooking. Apart from that, there was a big challenge of cafeteria infrastructure in the *labourer cafeteria*.

In this manual laborer’s cafeteria! I have already said…there is no chimney, no window, the surroundings are dirty; If the management clean this, then will I be able to provide better food.

The cafeteria operators mentioned that the employees preferred oily, spicy and fried food. They believed that people will not eat fruits because they are generally not eaten as a part of the main meal in Nepali culture, do not fit into the work breaks and are costly. The manual labourers preferred hot and fried food, and there was pressure that the curry should look ‘red’ (with addition of chili) to be considered to look appetising.

The manual laborers demand fried food. If I reduce the oil and spices, they (employees) will be angry.

When will they (employees) eat a fruit if I keep in the cafeteria? They want filling foods like rice and lentils, not fruit for lunch.

## Discussion

It is important to gather employee perceptions about healthy and unhealthy foods and learn about factors they view as barriers and facilitators to making healthy dietary choices in worksites. Findings from this study imply that a broad range of factors affect healthy eating in a factory-based cafeteria. Manual labourers described healthy and unhealthy foods in the context of hygiene, with freshly prepared foods being viewed as healthy. In addition, they identified fruits and vegetables as healthy foods. Packaged foods and soda were considered healthy despite lacking nutritional value. A commonly reported facilitator of healthy eating was the availability of affordable healthy food options in the cafeteria, which in turn depends on the commitment of the cafeteria management, knowledge and attitudes of the cafeteria operators, and adequate human resources. In addition, most participants cited that personal knowledge and attitudes contribute to making healthy food choices. Major barriers to healthy eating included unavailability of healthy food; difficulty changing eating habits; preference for fried food in the Nepali culture; and high cost of healthier foods. Cost of food in particular was a major concern for manual labourers who earn low wages.

Traditionally, Nepal has been burdened with food-borne diseases such as diarrhoea and typhoid.^24 25^ Thus, it is not surprising that participants were more concerned about hygiene compared with the nutritional value of foods. The reporting of fruits and vegetables as examples of healthy food is consistent with other studies reporting that consumers tend to agree that fruits and vegetables in particular are healthful.^26 27^ Despite the apparent knowledge of the importance of fruits and vegetables in a healthful diet, most Nepali people do not meet the recommended daily servings for fruits and vegetables.^28^ This suggests that interventions designed to increase fruit and vegetable intake in this setting should focus on factors influencing intake, such as eliminating barriers or increasing facilitators of healthful eating rather than on knowledge. The finding that the manual labourers believed that packaged foods and bottled soda were healthy reflects the low level of awareness of the low nutritional value of high-sugar beverages^29^ in Nepal.

The cafeterias in the factory depicted the clear socioeconomic and power hierarchy of the factory as there are three types of cafeteria with access to the three different levels of employees (manager, admin and labour). More options for healthy foods were found in managers’ cafeteria compared with labourers. In the labourer cafeteria, cheap foods were available because the external vendor who managed the canteen chooses the lowest priced foods available in the market. Special concern was mentioned about the reuse of cooking oil. There may be higher level of trans fat in the oil used for deep frying.^30^

A commonly discussed facilitator to healthy eating was the availability of healthy food options in the cafeteria. Both the employees and operators expressed the value of commitment from management in ensuring healthy eating in the cafeteria. It emerged in discussions with manual labourers that the clear demarcation of power dynamics led to manual labourers feeling unable to play a role in the decision-making process within the factory.

The main barriers to healthy foods reported by the manual labourers were a lack of healthy options, the prices of some healthier foods and the preference for fried foods. The lack of availability of healthy foods and the lack of time to prepare food were reported as two of the major barriers to healthy eating in other studies.^31^ Some of the participants emphatically stated during the discussion that they chose to eat in the cafeteria even if the foods were not healthy because healthier options outside of the cafeteria were more expensive. Cafeteria operators also indicated that the low-income level of manual labourers was a main barrier to providing healthier food options. Nestle and colleagues^19^ also reported that economic considerations may serve as barriers to healthy eating. Taste and personal preferences were also frequent barriers to healthful eating. Characteristics of foods such as taste, appearance and smell were reported as factors that most strongly influenced food choices in another study.^31^ Similarly, Glanz and colleagues^32^ found that taste and cost are the most influential factors of food choice. Likewise, in Tanzania, greater palatability, ease of storage, ease of preparation and variety of preparation methods were the main factors of food choice. In China, cultural barriers to accept brown rice were perception of rough texture, unpalatable taste and higher price. In Kuwait, factors influencing food consumption habits were taste, ease of preparation and cost.^33^

Occupational health research thus far is limited in Nepal.^34^ Although 85% of economically active Nepalese are engaged in farming or production,^35^ very little information is available on health and safety issues at these worksite and about work-related health problems. Most occupational health research in Nepal has been focused on pesticide use, needlestick injuries, back injuries and other occupational safety concerns.^36^ Recently, a study conducted among industrial workers in eastern Nepal reported a high prevalence of cardiometabolic risk. The industrial workers were young and had low educational attainment. One-third of the participants had high blood pressure, 47% had central obesity, 4% had diabetes, 44% had hypercholesterolaemia, 49% had hypertriglyceridaemia and 85% had dyslipidaemia. Furthermore, 40% of the participants were current smokers, 31% were hazardous drinkers, 97% had high salt intake and 38% did not consume any fruit.^37^ The findings highlight the need for an effective intervention to prevent cardiovascular disease in this population. The work described in this paper comprises a part of an effort to develop a dietary intervention in the worksite to mitigate the observed high cardiovascular risk prevalence.

Our study has several strengths worth noting. This is the first study to explore the facilitators and barriers to healthy eating in a cafeteria in a factory setting in Nepal. We purposely sampled the employees from two different strata, administrative staff and manual labourers, to represent socioeconomically diverse workers from the same worksite. Our FGDs achieved saturation^38^ when no new information was being shared by participants. We used an iterative process of data collection, conducted FGDs and IDIs, and then reviewed and improved the moderator’s guide after each discussion. We observed considerable variation in data and also uncovered some unanticipated information. The data collection and analysis was conducted in Nepali language by native speakers, which helped maintain a level of comfort and trust with the participants.

This study also has some notable limitations. First, it would have been valuable to stratify by body mass index (BMI) as overweight individuals might have different eating behaviours and perceptions compared with healthy weight individuals. Since we selected the participants randomly from each subgroup, it can be assumed that the views obtained in this study came from the adults who spanned the BMI spectrum. Second, this study did not include female participants. This study was only conducted among men because the factory only has male employees. It is possible that the perceptions and opinions would have been different among female counterparts due to the greater role Nepali women play in cooking and preparing foods. Third, the manual labourers had limited knowledge about healthy eating and were not aware about the relationship between diet and diabetes prevention. Hence, their discussion on facilitators and barriers was also limited by their lack of knowledge about healthy eating. Finally, since the employees had likely interacted with each other prior to the focus groups, the group process of discussion and interaction may have been influenced by peer relationships and each participant’s ability to express individual views.

Nonetheless, these findings are salient for the health promotion and prevention team working with the manufacturing factory. It is also critical to involve the employees including the administrative staff, manual labourers and cafeteria staff in planning intervention programmes to more holistically address their needs and create interventions that are both effective and sustainable. Understanding the perception, facilitators and barriers from the perspective of the employee and operator can aid in developing interventions to promote healthy eating.

### Implications for research and practice

Among the employees of a wire manufacturing factory in Nepal, healthy foods were commonly defined by employees in terms of hygiene rather than the type or nutritional quality of the food. However, fruits and vegetables were universally viewed as healthy regardless of hygiene. Availability of healthy food options at an affordable price, combined with an increased level of awareness and commitment from the factory management, can result in healthier food choices in the worksite. This suggests that interventions focusing on healthful, less expensive food preparation, or the selection of more convenient yet inexpensive healthful food, may help overcome the most common barriers in this population. These factors need to be addressed in designing appropriate cafeteria-based interventions in Nepal if they are to be effective in leading to sustained, healthy eating behaviours.

**Key messages**

**What is already known about this subject?**

Modifiable risk factors such as sedentary lifestyle, poor diet and excess body weight have a large effect on the risk of developing type 2 diabetes (T2D). Lifestyle interventions promoting healthy eating and physical activity have reduced T2D’s risk and related complications. In particular, a diet characterised by a low consumption of added sugar and refined grains, low in red and processed meat, and high intake of fruits, vegetables and whole grains has been shown to decrease the risk of T2D. As an important way to translate existing knowledge about T2D prevention efforts, worksite interventions can help promote healthy food choices, health education and social support.

**What does this study add?**

Research on worksite-based lifestyle interventions is lacking, particularly in low-income and middle-income countries such as Nepal. This study explored and informed the facilitators and barriers to healthy eating in a worksite setting in Nepal. In a worksite in Nepal, facilitator to eating healthy was availability of affordable health food options in cafeterias; and unavailability of healthy foods, difficulty in changing eating habits, the preference for fried foods in Nepali culture and the high costs of some healthy foods were the major barriers.

**How might this impact on clinical practice?**

The findings of this study can be used as a first step to designing lifestyle interventions to be implemented in the worksite.
